# Positive Clinical Outcomes for Severe Reported Pain Using Robust Non-Addictive Home Electrotherapy—A Case-Series

**DOI:** 10.3390/jpm13020336

**Published:** 2023-02-15

**Authors:** Anish Bajaj, David Han, Igor Elman, Panayotis K. Thanos, Catherine A. Dennen, Rajendra D. Badgaiyan, Abdalla Bowirrat, Debmalya Barh, Kenneth Blum

**Affiliations:** 1 School of Chiropractic, Cleveland University Health Sciences, Overland Park, KS 66210, USA; 2 Bajaj Chiropractic, New York, NY 10010, USA; 3 Department of Management Science and Statistics, University of Texas at San Antonio, San Antonio, TX 78249, USA; 4 Center for Pain and the Brain (P.A.I.N Group), Department of Anesthesiology, Critical Care & Pain Medicine, Boston Children’s Hospital, Boston, MA 02115, USA; 5 Cambridge Health Alliance, Harvard Medical School, Cambridge, MA 02139, USA; 6 Behavioral Neuropharmacology and Neuroimaging Laboratory on Addictions, Clinical Research Institute on Addictions, Department of Pharmacology and Toxicology, Jacobs School of Medicine and Biosciences, State University of New York at Buffalo, Buffalo, NY 14203, USA; 7 Department of Psychology, State University of New York at Buffalo, Buffalo, NY 14203, USA; 8 Department of Family Medicine, Jefferson Health Northeast, Philadelphia, PA 19114, USA; 9 Department of Psychiatry, South Texas Veteran Health Care System, Audie L. Murphy Memorial VA Hospital, Long School of Medicine, University of Texas Medical Center, San Antonio, TX 78229, USA; 10 Department of Molecular Biology, Adelson School of Medicine, Ariel University, Ariel 40700, Israel; 11 Centre for Genomics and Applied Gene Technology, Institute of Integrative Omics and Applied Biotechnology, Nonakuri, Purba Medinipur 721172, India; 12 Department of Genetics, Ecology and Evolution, Institute of Biological Sciences, Federal University of Minas Gerais, Belo Horizonte 31270-901, Brazil; 13 The Kenneth Blum Behavioral & Neurogenetic Institute, Austin, TX 78701, USA; 14 Center for Behavioral Health & Sports, Exercise, Psychiatry, Western University Health Sciences, Pomona, CA 91766, USA; 15 Institute of Psychology, ELTE Eötvös Loránd University, Kazinczy u. 23-27, 1075 Budapest, Hungary; 16 Department of Psychiatry, School of Medicine, University of Vermont, Burlington, VT 05405, USA; 17 Graduate College, Western University Health Sciences, Pomona, CA 91766, USA

**Keywords:** electrotherapy, H-Wave^®^, H-Wave^®^ home therapy program, reduced pain, non-addictive alternative, knee osteoarthritis, torn Achilles tendon

## Abstract

The North American opioid epidemic has resulted in over 800,000 related premature overdose fatalities since 2000, with the United States leading the world in highest opioid deaths per capita. Despite increased federal funding in recent years, intended to address this crisis, opioid overdose mortality has continued to increase. Legally prescribed opioids also chronically induce a problematic reduction in affect. While an ideal analgesic has yet to be developed, some effective multimodal non-opioid pharmacological regimens for acute pain management are being more widely utilized. Some investigators have suggested that a safer and more scientifically sound approach might be to induce “dopamine homeostasis” through non-pharmacological approaches, since opioid use even for acute pain of short duration is now being strongly questioned. There is also increasing evidence suggesting that some more robust forms of electrotherapy could be applied as an effective adjunct to avoid the problems associated with opioids. This 4-patient case-series presents such an approach to treatment of severe pain. All 4 of these chiropractic treatment cases involved a component of knee osteoarthritis, in addition to other reported areas of pain. Each patient engaged in a home recovery strategy using H-Wave^®^ device stimulation (HWDS) to address residual extremity issues following treatment of spinal subluxation and other standard treatments. A simple statistical analysis was conducted to determine the change in pain scores (Visual Analogue Scale) of pre and post electrotherapy treatments, resulting in significant reductions in self-reported pain (*p*-value = 0.0002). Three of the four patients continued using the home therapy device long-term as determined by a post-analysis questionnaire. This small case-series demonstrated notably positive outcomes, suggesting consideration of home use of HWDS for safe, non-pharmacological and non-addictive treatment of severe pain.

## 1. Introduction

More than 800,000 opioid-involved deaths have occurred since 2000, and the United States has the world’s highest number of opioid-involved deaths per capita. Although federal funding to address the opioid crisis has increased in recent years, opioid overdose mortality has increased as well. Deaths from opioid-involved overdoses were among the leading causes of death in 2020. Every 14 min, 150 million individuals are negatively affected by and suffer from painful conditions. Yearly, around 300 million narcotic prescriptions are filled, costing hundreds of billions of dollars. Some of these patients die from prescription overdose; others die from illicit Fentanyl laced products. It is well known that the consumption of potent narcotics to alleviate pain can result in higher tolerances and severe withdrawal symptoms within a relatively short period of time [1]. A website explaining the impact of chronic pain in the USA can be found at https://www.cdc.gov/mmwr/volumes/67/wr/mm6736a2.htm (accessed on 10 November 2022).

While sophisticated neuroimaging research performed by both NIDA and NIAAA, scientists are continuing to crack the neurobiological mechanisms of opioid use and misuse, the FDA along with the CDC are trying to reduce unwanted overdose-induced premature deaths through better MAT pharmaceutical approvals and prescribing guidelines. It is unfortunate that all we really have to offer patients dependent on opioids is the current Opioid Substitution Therapy (OST) approach. While some ASAM practitioners believe that administering opioids to OUD patients is considered treatment, we do not agree. Treating opioid addiction with powerful opioids like Buprenorphine in combination with Naloxone is not treatment [2].

While that is what we currently have according to the FDA, it is indeed a conundrum because it just “locks people into addiction” in spite of harm reduction [1]. While the CDC is trying to limit the overprescribing of analgesics for acute and even chronic pain, overdoses are still increasing [3]. One problem researched utilizing emotion detection technology clearly shows that legally prescribed opioids chronically induce a reduction in affect [4]. With this in mind, others have proposed that a more scientifically sound approach would be to induce “dopamine homeostasis” by considering non-pharmacological approaches to gently achieve this laudable goal [5]. There are a number of positive research studies regarding the clinical effects of, for example, the putative Pro-dopamine regulator KB220/KB220Z [6]. Along with this notion, Blum’s group developed the GARS test [7], to help identify DNA gene reward risk antecedents that could help pin-point brain reward neurotransmitter deficits as well as surfeits in what has been previously termed RDS [8,9]. Along these lines, it is important for the scientific community to embrace the idea that there is a simple way to assess and stratify “preaddiction” (similar to RDS) by coupling the validated RDSq29, GARS and Brain-, Spine-, and Mental- Health Screening Methods as the way to early identification of people at risk [10,11].

In understanding that OUD is indeed a brain disorder and a chronic life-time struggle—like diabetes—involving physiological, psychological and spiritual aspects, frontline modalities could benefit by the incorporation of alternatives like rTMS [12,13]. There is continual benefit from the use of both Cognitive Behavioral Therapy, Mindfulness, and other psychological approaches such as trauma therapy and even Unilateral Transcranial Photo biomodulation [14,15,16]. Certainly, there is good evidence that religiosity and spirituality have positive neurochemical benefits at the brain reward circuitry resulting in significantly reduced relapse [17,18,19,20,21,22,23,24,25,26,27,28,29,30]. Moreover, Opioid analgesia for acute painful conditions has come under increasing scrutiny with the public health crisis of opioid overdose, leading clinicians to seek nonopioid alternatives, such as nonsteroidal anti-inflammatory drugs (NSAIDs) and acetaminophen (paracetamol). The short-term use of opioids under close clinical supervision, such as in-hospital use of opioid analgesics for postoperative pain, may be appropriate, but even here, combination therapy or nonopioid therapy may be preferred. The use of opioids even for acute pain of short duration has been questioned. The ideal analgesic has yet to be developed, but effective pain control pharmacological regimens for acute pain are available [31].

- Most relevant to these important caveats to relieve pain is to embrace electrotherapy such as H-Wave^®^ device stimulation (HWDS), whereby there is significant evidence that its prescribed utilization could be a very useful tool instead of powerful opioids [32,33,34,35].

The aim of our study is to assess the potential analgesic and functionality induced by H-Wave device Stimulation using home therapy in chronic pain patients.

## 2. Materials and Methods

Study protocols were reviewed and approved by the PATH Foundation (NY) Institutional Review Board (IRB). The participants provided and approved written informed consent. The clinical studies were performed at Bajaj Chiropractic, P.C. in New York and in the homes of participants.

### 2.1. Protocols

These are 3 cases of knee osteoarthritis and 1 case of a previously torn Achilles tendon with knee osteoarthritis, all of whom engaged in home recovery strategies using H-Wave device stimulation. The pain protocol was a high frequency stimulation for at least 15 min with pad placement on the medial and lateral aspects of the knee joint. The lower frequency protocol involved pad placement above and below the knee joint, targeting circulation through the joint. Treatment protocols were anywhere from 15–30 min and administered a minimum of 3 times per week, up to 7 days per week. All results were captured over a 3-week period, with the average pain reduction being in the neighborhood of 75% (see table). Every one of the patients had been under previous and post chiropractic care, but for the period of this treatment/study, they were receiving just H-Wave at home (again, extension of care during the pandemic), resulting in a reduced load on the medical care system, reduced need for pain killers, reduced need for opioid-related pain relief, injections, and surgical interventions. It should also be noted that of these cases, 2 were already post-surgery, and 2 more were recommended for and, to this day, are pending knee replacement.

Prior to initiating H-Wave device Stimulation issues, specifically, in the extremities, we followed the usual protocol sequence for care: first, we addressed any spin-related issues (all explored extremity work starts with the spine)—including adjusting of possible stabilization of posture through the spine, whether it be range of motion of the spine or alignment of the spine; next, we worked on the feet, gradually working our way up to the knees. In this case group, those areas of correction were explored and had at least stabilized, reaching (at minimum) significant plateaus in results, leaving residual areas in need of additional rehabilitation considerations. 

Furthermore, home therapies were deployed in the absence of the ability to deliver what was considered non-emergent, non-life-threatening care during pandemic social distancing.

### 2.2. Characteristics of H-Wave^®^ Electrotherapy

The mechanisms of action of the H-Wave device stimulation (HWDS) assessed physiologically in a pre-clinical model included decreased edema [36]. Moreover, HWDS stimulates nitric oxide (NO)-dependent microcirculation increase as well as angiogenesis, resulting in tissue healing. The waveform and parameters of HWDS are distinct from other available electrical stimulation devices including TENS, NMES, etc.

The HWDS characteristics include:

- Low frequency (1–2 Hz) stimulation-induced contraction of smooth and skeletal muscle (red, slow-twitch) fibers lead to tissue loading while retaining the characteristics of low muscle force tension or non-fatiguing by avoiding tetanizing contractions.
- NO-dependent arteriolar vasodilation (revealed by rat studies).
- Bromouridine staining showed enhanced angiogenesis in repetitive stimulation in rats.
- Fluid shifts and reduced edema and protein clearance are caused by direct stimulation of smooth muscle fibers in the lymphatic vessels.
- A high frequency (60–70 Hz) mode can be used simultaneously to act intrinsically upon the nerve, affecting the function of the sodium pump within the nerve, to create a lasting anesthetic/analgesic effect.

Nonpharmacological alternatives are required to mitigate pain amid the current opioid crisis. It is well documented that opioid use can lead to respiratory depression [37]. Reduced respiratory drive and resulting low oxygen levels hinder systemic cellular function, let alone the healing of injured areas. Given our functional dependence on oxygen for survival, sustainable modalities such as H-Wave, which target supporting circulation, could play a broader role in both early injury intervention and recovery from chronic pain states. Over 18 studies including original articles, review articles, and abstracts are published in peer-reviewed journals illustrating the positive effects of H-Wave, including mechanism of action and pain relief [38,39,40,41]. Amidst our terrible opioid crisis, with several individuals losing their lives daily, alternatives to strong pain medications need to be adopted by the entire analgesia society.

Over the course of the past two decades, investigators have been increasingly keen in managing pain and restoring function by the use of electrical stimulation. One of the focal points of interest is the use of the H-Wave^®^ device [36,37,38]. The objective of the HWDS is to reduce chronic pain and inflammation. This can be achieved by [36]:

- Direct stimulation of the smooth muscles of lymphatic vessels and small-diameter skeletal muscle fibers by low-frequency (1–2 Hz) stimulation resulting in interstitial fluid shifts. Long rhythmical contractions of these particular muscles caused by HWDS lead to a decline in accretion of inflammation-associated proteins, an essential part of pain and associated disability in chronic injury or trauma patients.
- HWDS at high frequency (60 Hz) affects the function of sodium pumps in nerves leading to analgesic and/or anesthetic effects.
- NO-dependent stimulation of skeletal muscles induced by HWDS results in significant microcirculation increase, as evident from preclinical studies.
- Angiogenesis causes a profound and rapid increase in blood flow, which is seen in rat hind limbs post-repetitive HWDS.

Based on the data, it can be reasonably assumed that repetitive HWDS can reduce inflammation and aid in quicker healing and better recovery, owing to reduced protein accumulation in conditions such as post-operative rotator cuff reconstruction.

A meta-analysis by Blum et al., systematically reviewed the HWDS safety and efficacy for treating chronic inflammation of neuropathic and soft tissue pain. It included five studies linked to pain alleviation, decrease in utilization of pain medication, and improved function. Data were examined using the random-effects model, including correction to assess variability, study size, and bias in effect size [38]. This study utilized data from a total of 6535 patients [36,38]. Although there is a moderate-to-strong effect of the HWDS in offering pain relief, reduction in pain medication usage, and improved function as reported in this study, additional studies are warranted. The best result was noted for improved function, indicating that the HWDS can lead to a speedier return to work and other associated daily activities [33,34,35,42]. 

With these published works we decided to determine the efficacy in reducing pain in four patients with different pain issues utilizing H-Wave alone without coupling it with subluxation repair [38,39,43].

### 2.3. Statistics

We are aware of cohort limitations with this small population; therefore, we utilized simple statistical analysis and are cautious concerning overall interpretation of these results. 

### 2.4. Demographics (See Table 1)

The patient data table for this case group is inclusive of common population-based fac-tors such as age and sex, combined with the clinically relevant information including regions of interest, pain measures and functional improvements. 

**Table 1:** Demographic characteristics of the patients.

Age	68.8 ± 9.4 (range 58–78)	
Sex	Male 75%; Female 25%	
Region of Care	LE	100%
	Knee	75%
	Ankle	25%
	Foot	25%
Initial Pain	8.3 ± 1.0	
Post-treatment Pain	1.3 ± 1.3	
Delta/10	8.6 ± 1.1	
Taking Medicine	Yes 50%; No 50%	
Reduction of Medicine after Treatment	One patient reported 100% reduction, the other patient reported 75% reduction.	
Functional Improvement after Treatment	walk further	100%
	stand longer	100%
	more housework	75%
	greater ability to drive an automobile	50%
	lift more	50%
	sit longer	25%
	sleep better	25%

### 2.5. Statistical Analysis

Since there is a small number of participants (*N* = 4) for this study, it was decided to perform simple statistical analyses on these patients with (of course) some reservation as to outcome results. We used the statistical programming language CRAN R version 4.2.2 as well as Microsoft Excel to plot the figures and run a basic statistical analysis.

### 2.6. H-Wave Questionnaire(See Table 2)

The pain and function H-Wave questionnaire captured baseline and post-treatment pain scores as well as functional improvements in activities and treatment details in-cluding frequency and duration. 

**Table 2:** Pain and function H-Wave questionnaire.

What Conditions or Body Part Did You Utilize the H-Wave for?
If you were taking medication (for this condition) at the time you received your H-Wave, has H-Wave allowed you to decrease or eliminate the amount of medication taken?
If decreased, approximate by what percentage?
Has H-Wave allowed you to increase function or perform more activity than you could without it? If yes, please select all of the examples of things you are now able to do: No increased function; More family interaction; More housework; Walk further; Lift more; Greater ability to drive a vehicle; Sit longer; Sleep better; Stand longer; Other functions increased
Before using H-Wave, rate your average level of pain you were living with (0–10, 0-no pain, 10-extreme pain)
After using H-wave, rate your average level of pain you are living with (0–10, 0-no pain, 10-extreme pain)
How many times do you treat yourself per day?
How many days per week do you treat yourself?
How long is each treatment with the H-Wave? (Less than 30 min; 30–45 min; 45–60 min; 60+ min)

## 3. Results

### 3.1. Case Presentations

#### 3.1.1. Case One

The first patient (Case One) was a 64-year-old male at the time of the study; he came in with severe knee pain—particularly, in the left knee. He had a preexisting history of right knee pain with meniscus repair, which he had undergone surgery for. He had been told his knees were ‘bone on bone’, osteoarthritic, and likely in need of replacement. He was recommended to lose weight (as he was reported at 347 pounds) and to do physical therapy to better prepare for knee replacement. His other pertinent history was suffering from allergies, depression, type II diabetes, frequent urination with possible prostate implications, high blood pressure, nose bleeds, and sleep problems—including insomnia.

**Figure 1:**
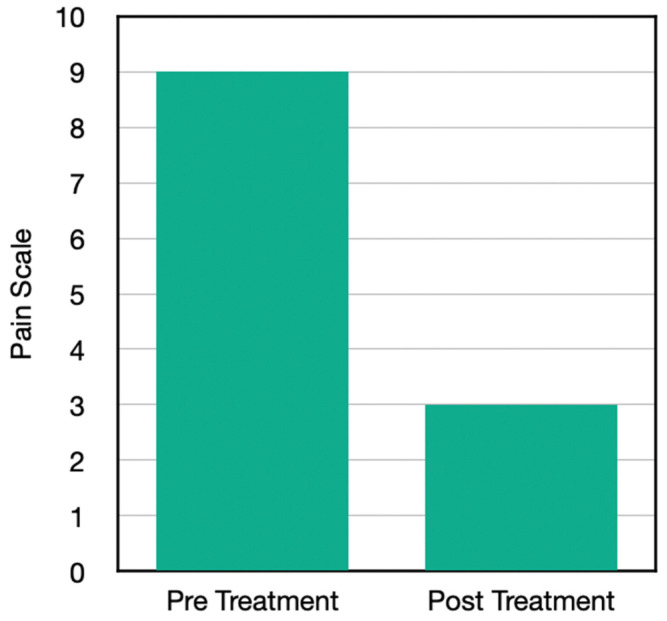
Pain scores before and after the H-Wave^®^ treatments.

Going into the chief complaint, his onset prior to reporting to the office was suffering with an excruciating pain (ranging from 8–10) 10 days prior to reporting to the office. He described it as a sharp pain, at times, while constantly aching. It was radiating into his lower back and into the legs. He had difficulty with bending the knee at all, lifting, physical activity, starting with walking. He was on unrelated medications, but he has historically tried over the counter medications, physical therapy, prescription medications, and surgery for these chronic knee problems. He also suffered from neck pain, but that was not the reason for this visit (Figure 1). 

In the intake examination, the patient presented with uncoordinated gait, misalignment, asymmetries, and tenderness at various locations in the spine. He presented with significantly reduced ranges of motion throughout the spine and lower extremity; he presented with significant guarding and muscle spasm in the spine; he presented with pain and restriction in normal spinal movement; he presented with pronation and increased q-angles bilaterally; he presented with pain and restriction in the lumbar ranges of motion; in the lower extremity, he had additional palpatory trigger points in the quadratus lumborum and gluteal. On palpation, he had positive response to compression in the lumbar, sacral, and dorsal regions. He had a short leg length in the prone position on the left. He had positive Lasegue’s on the right, positive Patrick Faber bilaterally, and difficulty with toe-in. Again, he was unable to perform other standing/bending tests due to significant leg restriction. 

His protocol with H-Wave included direct stimulation through the joint on high frequency for the first visit, which resulted in 70% pain reduction, after which he did homecare with both high and low frequency in order to sustain the results and improve overall circulation. His net effect was to go from an average of 9 on the pain scale down to a 3. Compared to previous interventions, this exceeded his expectations, and he was able to do that in protocols of 30 min or less.

#### 3.1.2. Case Two

Case Two was a 58-year-old male presenting with severe right Achilles pain. He had previous bilateral Achilles tears; he was not taking any medication at the time. He’d had incomplete recovery from those injuries and was significantly reduced in his functional capacity for exercise, with inability to run in addition to difficulty with prolonged standing, prolonged walking, and walking longer distances. He did this as a series of home care protocols and, like Case One, he took all these treatments without any other treatment, although he had had prior and post chiropractic care at our clinic. He also had complications of increased overweight, severe insomnia, problems with metabolism. He had tried soaking, icing, acupuncture, and had surgery but the ailments remained unresolved, resulting in a baseline constant pain (at minimum) of 4 out of 10, with sever exacerbations going up to a 9.

**Figure 2:**
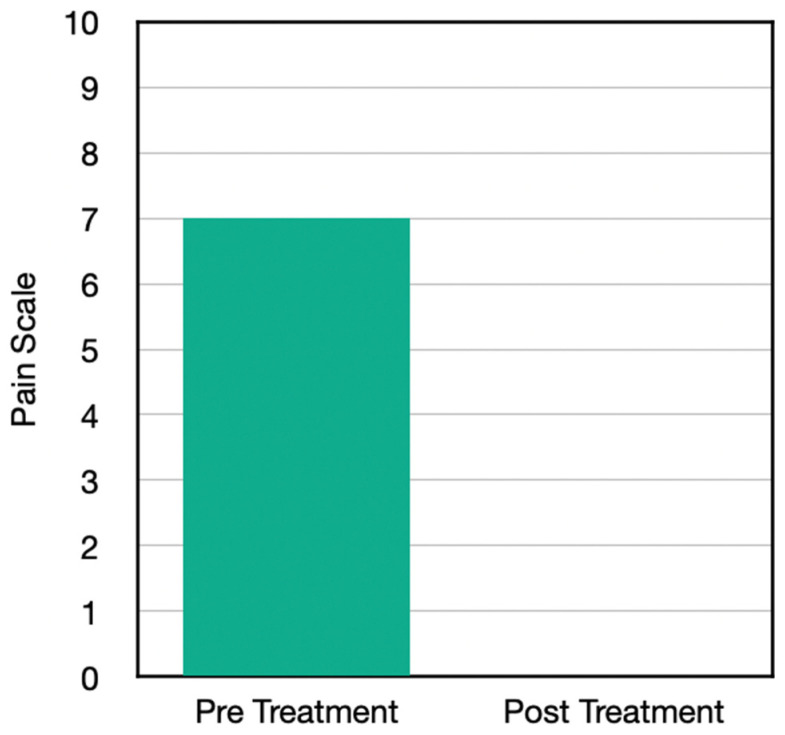
Pain scores before and after the H-Wave^®^ treatments.

After doing the H-wave device protocol on both the high and low frequencies, 2 times daily for two weeks, his pain went down to a 0. His pain was eliminated; he was able to do more house chores; he was able to walk further; he was able to lift more, and he had a better ability to drive, which included comfort in sitting. He was also able to sleep better and stand longer. This exceeded his previous expectation and was a significant improvement on top of the care he received with chiropractic only. This was all done by home care during the pandemic (Figure 2).

#### 3.1.3. Case Three

Case Three is a 78-year-old male with some history of foot injuries, lower leg injuries, and some chiropractic care for the spine but presented with knee pain—also (reportedly) due to osteoarthritis. He was not taking medication, but he was taking over the counter pain relief in the form of anti-inflammatories. He had not done injections, TENS units, nor electrical stimulation before; he had done physical therapy, chiropractic and home exercise, and his baseline pain started at an 8 out of 10. He did the high and low frequency of H-Wave protocol 3 days per week, ranging up to 30–45 min. After a period of just two weeks, his pain had gone down to 0.

**Figure 3:**
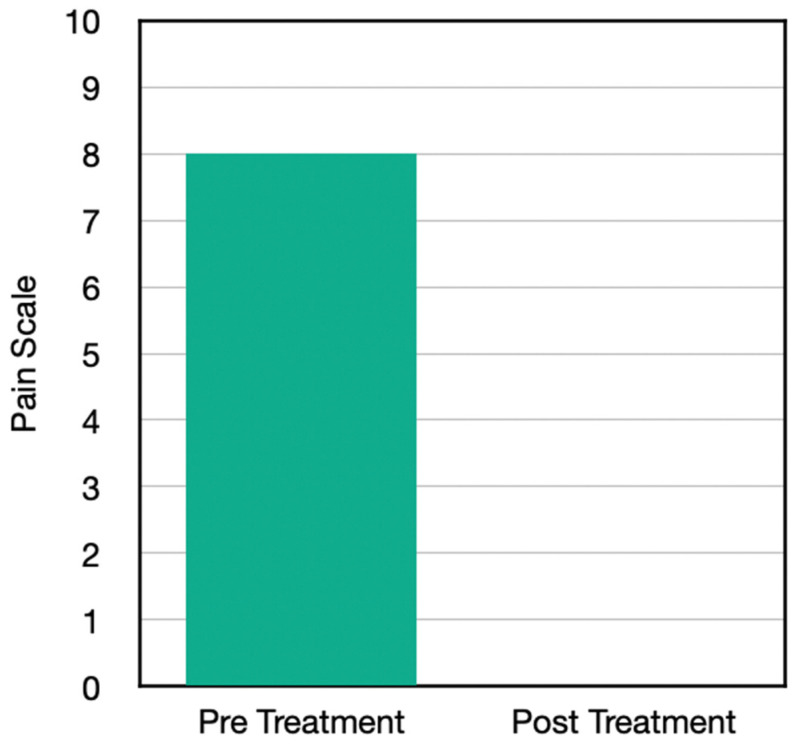
Pain scores before and after the H-Wave^®^ treatments.

His improvements included being able to walk further, lift more, sit longer, and stand longer. He had a better ability to drive an automobile for long distances. Previously, only certain sitting positions would bother him. These positions no longer bothered him; he was able to go down to a 0 on the pain scale, and he was able to decrease his usage of over-the-counter medications by 100% (Figure 3).

#### 3.1.4. Case Four

Case Four is a 75-year-old female with right knee pain due to bone-on-bone osteoarthritis. There was also a diagnosis of fluid in the knee, unlike Case One. The patient was taking over the counter anti-inflammatories, had done multiple injections, had been taking Advil quite frequently, and had done cortisone and gel. She had not recently done other rehab but had done electrical stimulation, physical therapy, and had received chiropractic for the spine before and after. During the treatment period, she started with (at the examination) an 8 out of 10 average pain score that ranged from 7–10 with exacerbations.

**Figure 4:**
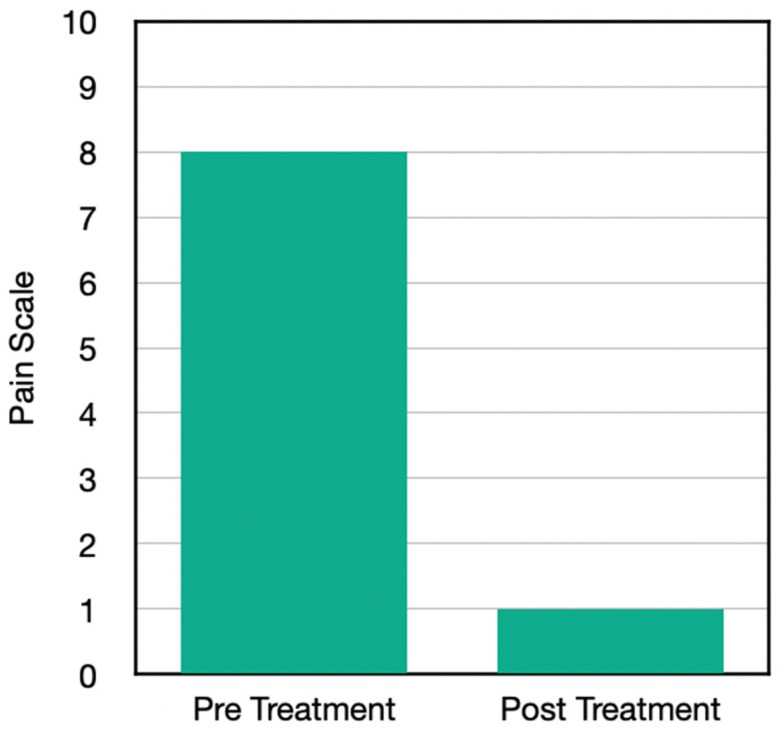
Pain scores before and after the H-Wave^®^ treatments.

After doing the H-Wave on high and low frequency for both pain management as well as circulatory support for 4–7 days per week, 30–45 min over a 3-week period, her pain level reduced down to a 1. She was able to resume exercise, which also expedited her recovery; specifically, she was able to walk further. She was able to lift more as a function of improved back pain, and she reduced her intake of over-the-counter pain medication by 75%. She was able to climb stairs better, had better exercise tolerance, and was able to get back to her normal recovery strategies (Figure 4).

Of particular interest, three of the patients continue to use H-Wave device home care while the other one does not. At this juncture we cannot assess if there are any long-term neuroplasticity effects in terms of positive neurotransmitter adaptations, a topic for future experimentation probably in animal models of pain. 

Results from the questionnaire post home H-Wave device stimulation resulted in a number of clinically important findings (Table 3). Case One with knee problems improved in the following clinical outcomes: walk further, reduction of pain, whereby pre-pain level was 9 and post H-Wave at level 3. Case Two with Achilles problems improved in the following clinical outcomes: more family interaction, more housework, walk further, lift more, greater ability to drive a vehicle, sleep better, stand longer, and reduction of pain, whereby pre-pain level was 7 and post H-Wave at level 0. Case Three with Knee problems improved in the following clinical outcomes: more housework, walk further, lift more, greater ability to drive a vehicle, sit longer, stand longer, and reduction of pain, whereby pre-pain level was 8 and post H-Wave at level 1. Case Four with knee problems improved in the following clinical outcomes: walk further, stand longer, and reduction of pain, whereby pre-pain level was 9 and post H-Wave at level 1. While each individual in the study showed significant reduction of pain and functional improvements, only 3 of 4 continued to utilize H-Wave for long term maintenance. One has continued maintenance homeware has included low frequency protocols to support ongoing circulatory needs (Case two). One has utilized the H-Wave for preventive maintenance (Case three). One has used the H-wave device at home for maintenance, continuing to avoid knee surgery as well as to recover from a wrist fracture involving release from care prior to complete recovery. The only member of the study that did not opt for maintenance care with H-Wave, unfortunately, underwent surgery and endured a lengthy recovery. 

## 4. Statistical Analysis Result

**Figure 5:**
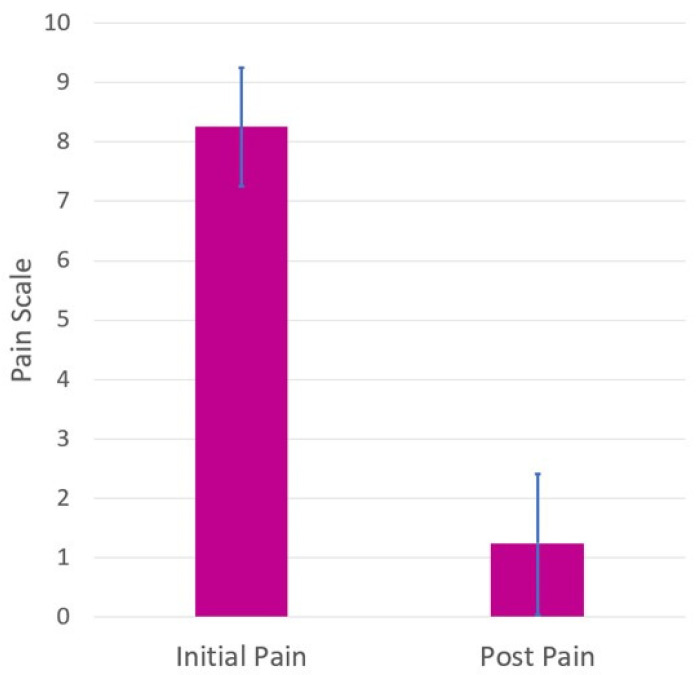
Average pain scores with standard deviations before and after the H-Wave^®^ treatments.

We conducted a simple statistical analysis to determine the change in the pain score of pre and post H-Wave device treatments in these four cases and found out that the pain score exhibited a significant reduction after the H-Wave treatment (*p*-value = 0.0002); see Figure 5.

## 5. Discussion

Our results showed a significant reduction of chronic pain. Our methods lead to relief from severe pain that has eluded the masses while improving function we associate with more sustainable and complete recovery. In the United States, abuse related to iatrogenic prescription drugs is the fastest escalating drug issue. Two major populaces at-risk in the USA related to prescription drug overdose are nine-million individuals reporting long-term medical opioid usage and five-million individuals reporting non-medical usage. Of the individuals who are prescribed high daily doses, 20% are receiving care under many clinicians. These individuals account for about 80% of overdose reports and are more susceptible to sharing the prescribed substances with others who use them without any prescription [4].

The central pathways that stem from the dorsal horn of the spinal cord to the medulla along with several genes and their biomarkers inhabiting the mesolimbic reward center of the brain play a role in controlling pain tolerance and sensitivity [44,45,46,47,48,49,50]. The presence of high muscle spindle densities in the spine, hands, and feet may underlie the importance of proper whole-body mechanoreception including balance, breathing and other essential posture-related functions. An important limitation is that these results do not involve RTC studies and require additional investigations; however, the results are encouraging. Albeit this limitation, applying H-Wave device stimulation to both the spine and extremities may simulate a more complete activation and recovery cycle in subluxated body regions presenting with fixation, misalignment or other movement deficiencies. Care modalities that compromise the healing process while guiding the focus of care on complete recovery of functional capacity over analgesia alone is certainly a laudable goal. 

Complexity of care suggests that no matter what region of interest needs repair, care will be complex in that it won’t address just a muscle, a ligament, a tendon, or any one specific tissue; it will be the process of reestablishing functional movement to each of the areas, especially when it comes to degenerative injuries. We address complexity of care by focusing on functional improvements. Each of the participants exhibited (as documented in the data table) significant functional improvements not otherwise achieved prior to going through the sequence of care, as outlined in this protocol. 

A common thread in the cases was the notion that they were presenting having reached maximum therapeutic benefit from traditional care and had “graduated” from physical therapy. By exploring mechanisms based in neurophysiology and the role of microcirculation, effective home care strategies were quickly identified to overcome gaps in traditional care. 

Since we were in an unwanted COVID pandemic, the four patients could not access a normal face to face with clinical professionals and, as such, that is why we explored H-Wave device stimulation—for both its ability to modulate pain and, in particular, its functions in resuscitating blood flow passively, and for its potential in establishing angiogenesis biologically. Our significant findings showing the benefit of reducing severe pain as presented in this pilot trial utilizing home H-wave device stimulation along with previous published works provide further rationale for continued investigation. This case series agrees with other previously reported data that evaluated both the efficacy and safety of the H-Wave device [32,33,34,35,36,38,39,40,41], but emphasizes the important utility of H-Wave device home therapy. While there are other useful therapeutic non-addictive analgesics, such as rTMS etc. [51], this small study provides additional anti-pain positive clinical metrics. In future studies, we can compare larger case groups to gain additional insights into the value of this approach to protocol building to address the complexity of severe chronic pain, whether lengthy [52] relatively short in duration.

It’s possible, we’ve pointed out again, that H-Wave device stimulation—with is potential for sustainable pain modulation and corrective mechanisms through passive movement assistance, tissue loading, and vascularization through NO-dependent angiogenesis—that these mechanisms, if they are consistently left over from traditional care that doesn’t always connect related areas like the lower extremities and major weight-bearing centers (like the spine) is also ignoring some of these essential functions related to balance and oxygenation, hence, the need for H-Wave early in care at the beginning of care, potentially reducing harm exposure in a much broader and impactful area of the population.

## 6. Limitations

Obviously, one major limitation is the small study cohort and, as such, interpretation must be restrained until more research is performed, especially with the H-Wave home therapy program. It is noteworthy that while the two patients taking pain medication actually reduced these medications by 75 and 100% is indeed laudable, more research must be completed before we could make a generalization concerning the role of H-Wave therapy in attenuation of pain medications. However, one could argue treatment bias related to non-systematic selection of the patient population matching age, ethnicity, type of pain, acute vs. chronic, length of time utilizing H-Wave, non-medicated patients, and blind placebo (sham device) RTC large population studies. Moreover, because of this small cohort, we could not assess the long-term benefits of H-Wave device stimulation as an inducer of, for example, neuroplasticity and prolonged pain reduction. In future studies, we are poised to combine H-Wave with subluxation repair, as described in our earlier papers [32].

Long-term exogenous opioids may harm some patients and for most others, it can be lifesaving. Currently, the OUD epidemic kills about 137 people daily in the USA and is problematic worldwide. It is estimated that there are about 2.1 million people in America that suffer from an OUD. It is now well-known that extended opioid use causes multiple irreversible changes to the brain, especially to both dopamine and opioid systems [45,46,47,48,49,50]. 

**Figure 6:**
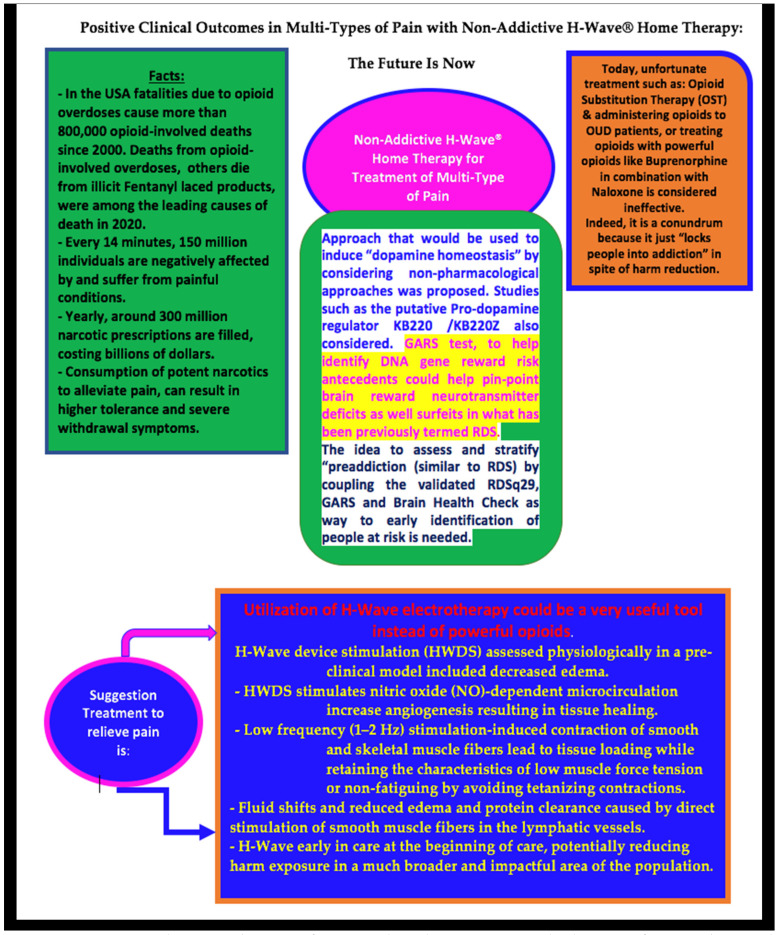
Schematic of positive clinical outcomes in multiple-types of pain with non-addictive and non-invasive H-Wave Home Therapy.

Below, we present a schematic summary of our model for comprehension of the readership (see Figure 6).

## 7. Conclusions

Similar to treatment resistant depression, there is a subpopulation that does not respond to standard OUD treatments, including MAT. Overdoses occur in naive and regular users, as adulteration, dosing, purity, and fentanyl content have wreaked havoc on the user and the illicit marketplace accustomed to being a parasite but recently more like a poison that not only takes over from the host but kills them. 

Certainly, we encourage additional research coupling H-Wave device stimulation with subluxation repair, which together provide a more complete system of recovery with a wider bandwidth of healing that addresses commonly overlooked movement-driven microcirculation and resuscitation of functional neurological pathways. Our group has work in progress on a larger subject base we hope will add more perspective to these findings. This small but significant study showing a positive outcome with home use of H-Wave provides rationale for the potential of H-Wave device stimulation to treat severe pain as a non-pharmacological, non-invasive and non-addictive effective and safe alternative.

## Figures and Tables

**Table 3:** Questionnaire Results.

Question	Case 1	Case 2	Case 3	Case 4
What conditions or body part did you utilize the H-Wave for?	Knee	Achilles	Knee	Knee
If you were taking medication (for this condition) at the you received your H-Wave, has H-Wave allowed you to decrease or eliminate the amount of medication taken?	No	No	Yes	Yes
If decreased, approximate by what percentage?	N/A	N/A	100	75
Has H-Wave allowed you to increase function or perform more activity than you could without it? If yes, please select all of the examples of things you are now able to do:	Yes	Yes	Yes	Yes
More family interaction	No	Yes	No	No
More housework	No	Yes	Yes	No
Walk further	Yes	Yes	Yes	Yes
Lift more	No	Yes	Yes	No
Greater ability to drive a vehicle	No	Yes	Yes	No
Sit longer	No	No	Yes	No
Sleep better	No	Yes	No	No
Stand longer	Yes	Yes	Yes	Yes
Other functions increased	N/A	N/A	N/A	Climb Stairs; Exercise; Balance
Before using H-Wave, rate your average level of pain you were living with (0–10, 0-no pain, 10-extreme pain)	9	7	8	9
After using H-Wave, rate your average level of pain you are living with (0–10, 0-no pain, 10-extreme pain)	3	0	1	1
How many times do you treat yourself per day?	1	2	1	1
How many days per week do you treat yourself?	3	7	3	4
How long is each treatment with the H-Wave? (Less than 30 min; 30–45 min; 45–60 min; 60+ min)	<30	30	30–45	30–45
Current use of H-Wave	No	Yes	Yes	Yes

## Data Availability

The data is contained within the manuscript.
